# How Does the Mental Health Lived Experience Workforce Describe and Enact Our Discipline? A Narrative Literature Review

**DOI:** 10.1111/hex.70812

**Published:** 2026-08-09

**Authors:** Cath Roper, Shibs Sharpe, Leanna Azoury, Nina Joffee‐Kohn, Natasha Swingler, Puneet Sansanwal, Leah McKenner, Cat Van Remmen, Bridget Hamilton

**Affiliations:** ^1^ Centre for Mental Health Nursing, Department of Nursing University of Melbourne Melbourne Victoria Australia

**Keywords:** consumer, Indigenous well‐being approaches, leadership, lived experience, mental health reform, mental health services, peer, service‐user, workforce

## Abstract

**Introduction:**

Worldwide, mental health systems continue to grapple with providing recovery‐oriented services as part of broader reform efforts. Critical to the objective of transforming mental health services is the employment of a rights‐based, non‐clinical lived experience workforce. Vital to undertaking the roles, and supporting this workforce, is a comprehensive understanding of the knowledge and practices of the discipline, particularly in situations where rapid growth may see this workforce developing in an ad hoc way. The aim of this review was to determine how members of the mental health lived experience workforce describe and enact our discipline, by synthesising evidence and illustrations.

**Methods:**

A narrative review methodology employed searches for international peer reviewed literature and policy documents related to lived experience work. Resources required at least one mental health lived experience author. We used a deductive approach to the categorisation of three designated mental health lived experience role types: service delivery (such as peer support); leadership; and Indigenous. An inductive approach was then taken to identify the knowledge and practices informing the three role types. Relevant texts were allocated amongst the research team, independently read, coded and themed, followed by team discussions to reach consensus where there were conflicts.

**Results:**

After full text reading, we identified 25 resources. There were many more resources about the knowledge and practices of lived experience direct service delivery than about leadership roles. Three themes emerged from the knowledge and practices for each role type. For direct service roles knowledge was: having been there; knowing helpful qualities of relationships; and knowing how to use your own lived experience intentionally. Practices for direct service roles were: working relationally, sharing lived experiences in a meaningful way and working in a values and rights‐based way. Knowledge required for leadership was: ethical decision‐making; being informed by collective/shared history and knowing how to create change. Required practices were: transforming services from within; embedding the lived experience workforce and championing justice. Themes describing Indigenous knowledge were: challenging exclusively Euro‐centric modes of treatment, truth‐telling and self‐determination. Emerging themes on practices of designated Indigenous lived experience workforces were: relational and community approaches, trauma informed, and culturally sensitive and safe practices.

**Conclusion:**

Articulation of the knowledge and practices underpinning the lived experience discipline is required for this workforce to be confident in the scope, purpose and history of their roles and for organisations to successfully establish them and provide effective supports. For Indigenous workers in designated lived experience roles, approaches to wellbeing and cultural practices cannot be separated from the way that the roles are carried out. Embedding Indigenous leadership, knowledge and practices would strengthen the human rights, justice‐based and non‐clinical nature of these roles, is a necessary response to colonial violence and would guide workforce development for other LE staff, mental health professionals, and mental health service development.

## 1 Introduction

Unlike physical health, which protects the principle of informed consent to treatment, specific legislation enables public mental health services to authorise detention, administer treatment without consent, and use restrictive practices. Such legislation operates across inpatient and community settings, invoking human rights issues. Over‐reliance on biomedical approaches to human distress is linked with failure to account for multiple ways people make sense of experience, downplaying the role of social and cultural context and increasing coercion and stigma (World Health Organisation and the United Nations, 2023). As mental health services globally grapple with human rights concerns, and the transformation to recovery‐oriented services, the further growth of a lived experience workforce (LEW) is timely, as our discipline is deeply rooted in human rights as a matter of principle and practice [1, 2].

The LEW has roots in a socio‐political movement sometimes referred to as the consumer/survivor movement (Beresford & Rose, 2023; Chamberlin, 1990 [3]; Scholz, 2022). In the 1960s and in common with other oppressed groups, people who had been treated and hospitalised without consent identified a loss of human rights and citizenship. Such movements were rights‐based, committed to social justice, promoted anti‐discrimination and a recognition of connections between political power, structural issues and inequities (Beresford & Rose, 2023 [4];). The movement also featured self‐advocacy, highlighting issues of intersectionality, appreciating the impact of social determinants in people's lives, and opposing all forms of coercion (Beresford & Rose, 2023). Such a heritage means a knowledge focused on human rights and an activist stance in practice is intrinsic to LE work [5].

### 1.1 Definition of Lived Experience (LE)

We use ‘lived experience’ to mean experiences commonly cast as mental ill health and/or using mental health services or not being able to access mental health services when needed.

### 1.2 Definition of Lived Experience Workforce (LEW)

For the purposes of this review, Lived Experience roles in mental health ‘are distinguished from clinical roles by the use of lived expertise and experiential knowledge gained through experiences of diagnosis, service use, and personal recovery from mental health challenges for the purpose of helping others and contributing to system change’ [6] (p. 1445). For our purposes, the LE workforce (LEW) does not refer to employment of people with lived experience of a caring role, although when we refer to Indigenous contexts, we adopt the following definition:

A lived experience recognises the effects of ongoing negative historical impacts and or specific events on the Social and Emotional Wellbeing of Aboriginal and Torres Strait Islander peoples. It encompasses the cultural, spiritual, physical, emotional and mental wellbeing of the individual, family or community [4].

(p.13)

Nor does it describe a person who has lived experience, but is not employed in an LE designated role, such as clinical staff with lived experience. That is, LEW denotes roles where lived experience is part of the job title and an essential criterion of a position description. The practice of employing a person into a designated LE role, who does not have the necessary expertise and grounding in the LE perspective, or employing a person who has lived experience but is working in a non‐designated role (such as social worker or psychologist) has been critiqued in the literature as attenuating the authenticity and expertise required to intentionally use LE perspective [7]. Further, an LE worker is not a ‘para clinician’ [8] involved in assessing service‐users, reading clinical notes, or attending clinical meetings (unless requested by the service‐user).

The aim of this review is to define and illustrate the LE discipline by synthesising contributions from the literature regarding knowledge and practice of each of three role types: direct service roles, leadership and Indigenous LE roles in mental health. We use the term LE ‘discipline’ in much the same way as social work or psychology are referred to as a discipline, comprising their own distinctive body of knowledge, philosophy and practices [6, 9].

Decades of debate, thinking, study and collective work have resulted in particular ways of regarding our experiences and ourselves [7]. LE work is not focused on diagnoses but typically on a commonality of oppression, loss of citizenship, powerlessness and sharing parts of these experiences in ways that are useful to others [7]. Personal recovery, growth and learning are other elements of first‐hand experience and mutually shared knowledge [10, 11, 12, 13]. When we examine the enacting of our discipline, we mean how the discipline is applied. The distinctive knowledge and practices of this discipline apply across the range of settings that the LE workforce work within including but not limited to mental health acute and community services, non‐government sector services and Emergency Departments. Application of the LE discipline occurs through all role types such as governance, leadership roles, academia and service delivery, however, the scope of roles vary [14].

Previous research has identified discrepancies between what LE workers understand their roles to be and what services understand LE roles to be [15]. Organisations and members of the LEW need clear understanding of the distinctive knowledge and practices informing the work, which then informs its scope of practice and helps this workforce to thrive. Difficulties arise when there is ad hoc organisational support, role confusion, lack of consistency and unfamiliarity with what LE perspective knowledge and practices entail. In such situations, clarity on what is unique about LE roles may be lost [16], and people in LE positions may be set up for failure [1], limiting the effectiveness and value of LE roles in mental health services [6, 8, 17].

The LE discipline was identified in the literature as vulnerable to organisational expectations that are beyond the LE workforce's scope of practice or a misunderstanding of the roles [17]. Role isolation may increase uncertainty for LE workers about what they are supposed to be doing [17]. Rapid growth of this workforce, without investment in discipline and practice supports developed by those engaged in the work, is likely to exacerbate such challenges. Lived experience roles must retain their distinctiveness as rights‐based, non‐clinical roles. Policy decisions, workforce frameworks and guiding documents must consider the support needs of the LE workforce, including workplace supports, training and leadership development. LE expertise must lead such initiatives to maintain fidelity to the LE discipline [18]. Unless their distinctive value is well understood, opportunities to maximise the unique foundations of this workforce will be lost.

Although currently drastically underdeveloped, designated Indigenous lived experience roles in mental health offer much needed non‐clinical, relational, and human rights‐informed approaches. Embedding designated Indigenous leadership would strengthen the effectiveness of an Indigenous workforce and that of other LE roles, health professionals and mental health services and is a necessary response to colonial violence. Promoting this leadership is important because research shows that for Indigenous people, white colonisation is embodied in everyday lived experiences of interpersonal and institutional racism including in health services (Askew et al., 2021).

## 2 Methods

### 2.1 Positioning Statement

Core authors of this review were LE researchers working in academia, with support from a non‐LE research supervisor. An Aboriginal LE researcher on the team led the analysis and theming of Indigenous resources. Through ongoing discussions and review, they played a key role in shaping the direction of the study by foregrounding Indigenous knowledge and ways of knowing often overlooked in the discipline.

To develop an understanding of how LE workers describe the knowledge and practices of their discipline, we conducted a narrative review of studies with at least one LE researcher. A narrative review was appropriate for our topic as it can include a range of resource types, such as grey literature (articles that are not peer‐reviewed, e.g. conference proceedings, government reports) as well as different methodologies and/or topics (Cronin & George, 2020). Accessing grey literature was vital for this review because much of the LE knowledge is not located in formal peer‐reviewed literature. Our review provides a novel synthesis of the gathered source material and draws on the expertise of the LE research team [19].

We used a deductive approach to the broad categorisation of our data, dividing it into three domains of designated mental health lived experience role types: service delivery; leadership; and designated Indigenous lived experience roles in mental health. An inductive approach was then taken to identify within the studies the knowledge and practices informing the three broad role types. Our approach allowed us to highlight the impact of missed opportunities for Indigenous leadership and knowledge practices to be embedded in the LEW so far, which also raises questions of how the violence of colonisation has and should be responded to in this context.

Two lived experience researchers conducted the initial electronic database searches in PubMed, PsycINFO, and CINAHL using the terms ‘mental health’ and ‘consumer’ OR ‘lived experience’, OR ‘mental health service user’ OR ‘peer’ AND ‘workforce,’ OR ‘role’, OR ‘workforce development’ OR ‘mental health reform’ OR ‘mental health services’ as well as ‘Indigenous’ OR ‘First Nations’ OR ‘Aboriginal and Torres Strait Islander’ AND ‘Mental Health’ AND ‘lived experience workforce’, OR ‘peer workforce’. Google scholar searches with the same terms, hand‐searching and citation tracking were used to identify other seminal sources that had not yet been located from our initial searches, in particular, designated Indigenous lived experience workforce perspective [20].

All relevant resources were loaded into Covidence, as an online shareable tool for all researchers. Duplicates were removed. The same two LE researchers screened titles, then the seven‐member team screened abstracts, and any conflicts were discussed with the team until agreement was reached. There were 33 resources after abstract screening. Following this, we created an online spreadsheet, accessible by the whole research team, and allocated the resources for independent review between the five lived experience researchers for full‐text reading (two of the original research members had left the team at this point). A further eight sources were excluded for not meeting our inclusion criteria, see Table 1 below, following full text reading and team discussion, leaving us with 25 included resources.

**Table 1:** Inclusion and exclusion criteria.

Inclusion criteria	Exclusion criteria
Published between 2010 and 2025	Benefits of LE perspective to other disciplines
English language	If the research was clinician dominated
At least one LE author	Research concerning lived experience but not in a workforce context
Focus on paid LE workforce roles in publicly funded mental health services and non‐government organisations	Documents where LE roles referred to carer or non‐designated roles (except in the case of Indigenous research/policy).

**Figure 1:**
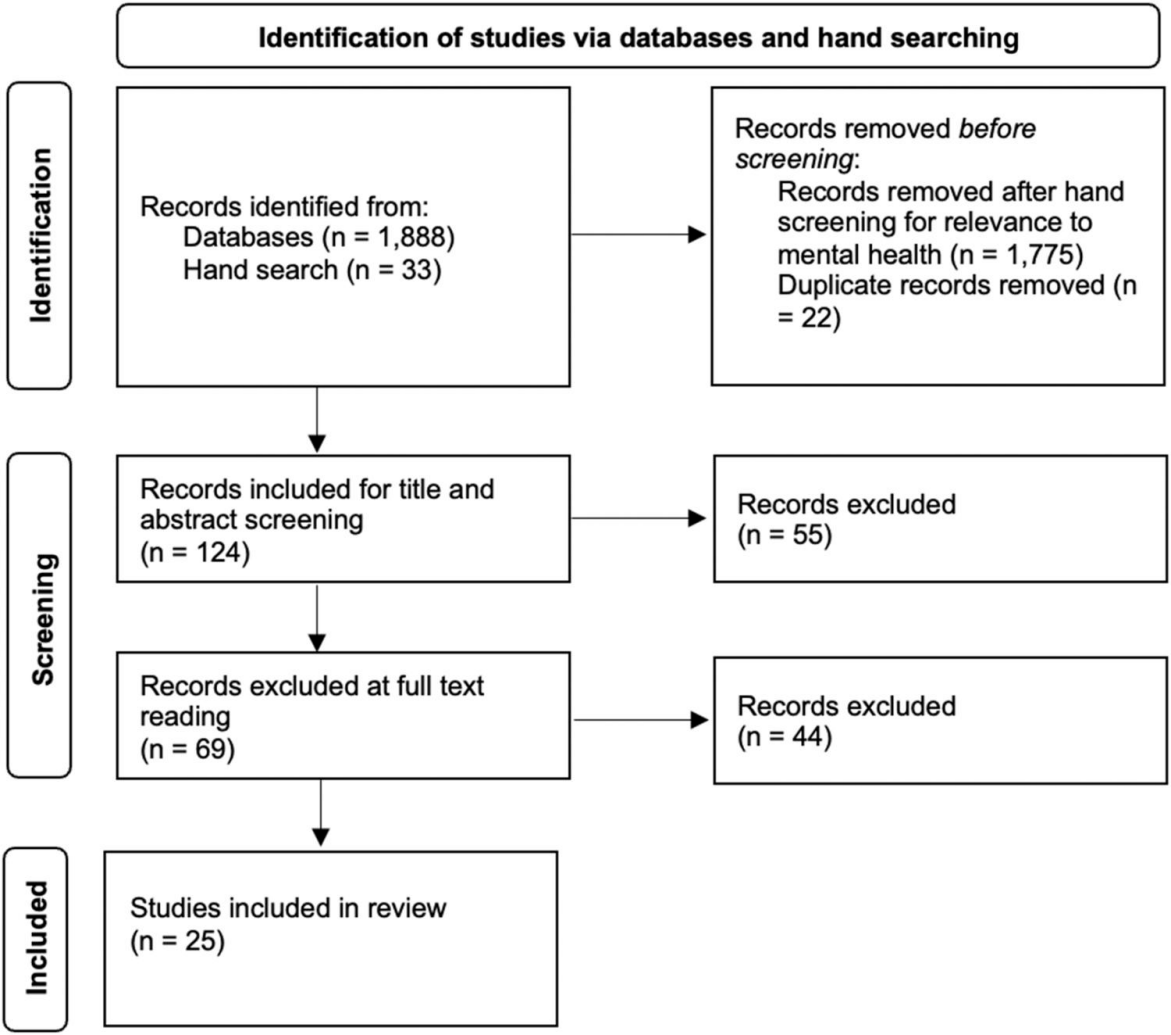
Identification of studies via databases and hand searching.

All the LE researchers participated as reviewers and in the resolution of conflicting decisions which occurred in research team meetings with a consensus approach to decision‐making. We used Mendeley software to automatically create our bibliography and to organise, read, and annotate each full text independently. Each independent reviewer also recorded the themes emerging from their reviewed articles onto an online Padlet page, providing access for all reviewers to share emerging themes from their reading. No form of quality appraisal of included sources was undertaken, rather, the articles were read and summarised for their relevance to our topic, and we used a descriptive approach to the data. Figure 1 below shows the process used for identification of studies via databases and hand searching.

## 3 Results

Table 2 below shows the full set of included results. After full‐text reading we had 25 resources: 10 reflecting direct service roles: six peer‐reviewed journal articles, one LE policy Framework from Western Australia, one book chapter, one PhD thesis summary and a brochure on the competencies of peer support. There were eight resources on LE leadership roles with a rich lineage of scholarship evident, including a research project from 1997 which was instrumental in the development of LE consultant roles in Victoria, Australia, aiming to transform mental health services at a systemic level and be a conduit between service users and clinicians. In terms of designated Indigenous LE roles, we identified a Western Australian Aboriginal and Torres Strait Islander Framework. In a subsequent google search, we identified six further resources on Indigeneity and working in designated LE roles in mental health services; two from Canada, two from New Zealand, one more from Australia and one from the US. In Table 2 columns describe aims, methods, results, type of article, eg if the literature was peer reviewed or grey, rows show if the resource was about direct service provision, leadership or Indigenous LE knowledge and practice.

**Table 2:** Summary of included literature.

Author/s	Year of research	Country of origin	Title	Aim of research	Method	Results	Type of resource
Direct service roles such as peer support							
Jacobson, N., Trojanowski, L. & Dewa, C.S.	2012	USA & Canada	What do peer support workers do? A job description	Critical ingredients of peer support work – what and how peers do their work and proposing a job description for the peer support role.	Participatory evaluation of a peer support programme at a psychiatric tertiary care facility to specify the work that peers do.	The main types of direct and indirect work are explored.	Peer reviewed journal article
Peer support Canada	2025	Canada	Required competencies of peer supporters	Articulate competencies for peer support workers	Developed by Peer Support Canada in collaboration with peer support leaders	11 competencies for peer support workers are outlined in this brochure	Brochure
Byrne, L., & Roennfeldt, H	2024	USA	A Model for Understanding Lived Expertise to Support Effective Recruitment of Peer Roles	The study explored participants’ views on the essential knowledge and skills derived from lived experience to inform the design of peer roles and support effective recruitment.	Qualitative study. 132 participants including managers, peers and colleagues were asked about the essential knowledge and skills of peer roles to inform recruitment and role design.	Aspects of LE comprise three domains: life‐changing individual experiences and impacts; identification as a peer and understanding and application of the collective peer thinking and values; and ultimately, lived expertise, a unique, experientially developed knowledge base and set of skills that can benefit others.	Peer reviewed journal article
Wyder, M., Roennfeldt, H., Parker, S., Vilic, G., McCann, K., 1 Ehrlich, C., and Dark, F.L.	2020	Australia	Diary of a Mental Health Peer Worker: Findings From a Diary Study Into the Role of Peer Work in a Clinical Mental Health Setting.	This study explored the experience of paid peer support workers integrated within a clinically‐operated community‐based residential rehabilitation service.	A general inductive approach was taken in the analysis of 36 diaries completed by a newly employed peer workforce. These diaries focused on what they viewed as significant interactions in fulfilling their role. Vignettes illustrated key themes.	Peer support workers described their work as flexible, responsive, and adaptable to the resident's needs and believed their roles brought a different lens to interactions on the unit, fostering more inclusive and personal ways of working.	Peer reviewed journal article
Oborn E, Barrett M, Gibson S, Gillard S.	2019	UK	Knowledge and expertise in care practices: the role of the peer worker in mental health teams	The study focused on how LE contribute knowledge and expertise to mental health care within multidisciplinary healthcare teams.	A 2 year longitudinal study undertaken by a multidisciplinary team conducted 91 interviews with peer workers and other stakeholders comparative case study design.	The study raises the question of how subjective forms of expertise might best be incorporated into multidisciplinary care.	Peer reviewed journal article
Government of Western Australia Mental Health Commission	2022	Australia	Lived Experience (Peer) Workforces Framework (WA)	Aim: how an LE workforce is part of bridging inequities, and designing and delivering human rights based, socially just, practices. How to build, embed & sustain diverse LE workforces across the mental health, alcohol and other drugs and suicide prevention sectors.	Policy document. Consultation and engagement undertaken with multiple stakeholders.	LE understood as an expertise & discipline, our values and principles, scope of roles, ways of working, history and has SEWB & Indigenous perspectives woven throughout. Articulates roles within LEW.	Policy document – grey literature
Mead, S., & Filson, B.	2017	USA	Mutuality and shared power as an alternative to coercion and force.	To show that relationships and connection can avoid coercion in mental health services	Opinion piece that describes examples of using relationship to avoid coercion	Not an example of ‘real world’ research, but a description of practices that can be used to avoid coercion	Opinion piece, non‐empirical with practice examples, Peer reviewed journal article
Meagher, J., and Naughtin, G.	2018	Australia	Scope, Role and Contribution of Peer Work: derived, synthesised and analysed from selected peer work literature. In: Meagher J, Stratford A, Jackson F, Jayakody E, Fong T, [eds]. Peer work in Australia: A new future for mental health.	This chapter aims to define peer support work, its value and the scope of roles.	The chapter provides a synthesis of information about peer work gathered from ideas, concepts and findings from the literature and policies.	Key content includes: the goals of the roles; the values and principles underpinning peer work; the contribution that peer support makes; the evidence base and history of peer work.	Book Chapter
Watson, E.	2017	UK	The mechanisms underpinning peer support: a literature review	To review the published research literature relating to the process of peer support and its underpinning mechanisms to better understand how and why it works.	A scoping review of published literature identified studies relating to peer support mechanisms, processes and relationships. Studies were summarised and findings analysed.	Findings: five mechanisms underpin peer support: lived experience, love labour, the liminal position of the peer worker, (both service user and mental health worker), strengths‐focussed social and practical support, and the helper role).	Literature review published in a peer reviewed journal
Prowse, C.	2022	Canada	Troubling Peer Support Institutionalisation: A Mad Institutional Ethnography; Or, Everyday DocumentationDe/Valuing, & Values Work in Institutionalised Peer Support	The aim of this research was to explore peer workers’ everyday work and the tensions they felt in relation to peer support institutionalisation.	Article from a PhD thesis using Institutional ethnography to explore how the work of peer supporters is shaped by institutional forces. Pieces of ‘text’ were explored (e.g., legislation, policies, discourse) that impact their work.	Documenting and case noting in a clinical setting is troubling for peer work, breaches privacy and principles of consent. There is a disjuncture between how confidentiality and consent are known to peer workers and clinical staff.	Summary from a PhD thesis
Leadership roles							
Bennetts, W., Pinches, A., Paluch, T., Fossey, E.	2013	Australia	Real lives, real jobs: Sustaining consumer perspective work in the mental health sector	Explores issues faced by those undertaking LE roles in Victoria's mental health system	Qualitative research interviewing 24 people working in LE roles	Five themes: the essence of consumer work; the dollars; pathways – education, training, supervision and mentoring; the workplace; and leadership	Peer reviewed journal article
Edan, V., Sellick, K., Ainsworth, S., Alvarez‐Varquez, S., Johnson, B., Smale, K., Randall, R., & Roper, C.	2021	Australia	Employed but not included: the case of consumer‐workers in mental health care services.	How do mental health consumer–workers experi‐ence employment, HRM and the organisations in which they work?	77 consumer workers’ experiences – mixed methods survey and indepth interviews using a co‐design approach	HR scholarship in the context of ‘mental illness’, needs to draw on other disciplines and engage with alternative perspectives and co‐design approaches are recommended.	Peer reviewed journal article
Byrne, L., Stratford, A., & Davidson, L.	2018	US	The global need for lived experience leadership.	Mental health LE roles risk being coopted without LE leadership which is needed to guide Its growth and development	Opinion piece	Explores the value of LE leadership in mental health services and the need to maximise.	Peer reviewed journal article
Roennfeldt, H., & Byrne, L.	2021	Australia	Skin in the game: The professionalisation of lived experience roles in mental health.	The arguments for and against professionalisation of the LE workforce are explored to identify the risks, benefits, and considerations.	Theoretical research	LE roles are a distinct professional discipline and other disciplines can advocate for its distinctiveness to avoid co‐option.	Peer reviewed journal article
Sinclair, A., Fernandes, C., Gillieatt, S., & Mahboub, L	2024	Australia	Peer work in Australian mental health policy: What ‘problems’ are we solving and to what effect(s)?	This research aims to re‐imagine peer work and inclusion that would achieve self‐determination and social justice.	Theoretical research	Investigates way peer workers are included in Australian mental health policy, and effects on peer workers and their practice.	Peer reviewed journal article
Stewart, S., Warner, T., Edan, V., Makuvachuma, D., Kennedy, H., Roper, C., Scholz, B., and Griffiths, S.	2023	Australia	Power of Self as the Resource and the North Star	Produce an account of the defining features of lived experience leadership, as understood by those engaging in its practice.	Qualitative, peer reviewed. Interviews conducted with 19 people defined by their peers as lived experience leaders, asked to discuss lived experience leadership and related concepts such as authority, power and influence.	LE leadership is distinct from other forms of mental health leadership, founded upon use of power of self; grounded in reflexive orientation to self & collective from both experiential and systems‐informed perspectives.	Peer reviewed journal article
Epstein, M., and Shaw, J.,	1997	Australia	Developing Effective Consumer Participation in Mental Health Services: The Report of the Lemon Learning Project	Report on educational strategies developed by LE for mental health clinicians & supporting the introduction of on‐staff LEW in acute settings.	Grey literature. Consultations with LE and leadership of LE researchers.	LE only spaces support the work of on‐staff LE consultants. Facilitated deep dialogue can enhance clinicians’ understanding of LE roles.	Grey literature
Loughhead, M., Hodges, E., McIntyre, H., Procter, N.G., Barbara, A., Bickley, B., Harris, G., Huberand, L., Martinez, L.	2023	Australia	A model of lived experience leadership for transformative systems change: Activating Lived Experience Leadership (ALEL) project	The model described in this paper aimed to increase the recognition and understanding of lived experience leadership in mental health and social sectors.	Participatory action research methodology and cocreation methodology. Focus groups with LE leaders, interviews with sector leaders and a national survey of LE leaders provided the basis of qualitative data, interpreted via an iterative, shared analysis.	The model frames LE leadership as a social movement for recognition, inclusion and justice comprising six leadership actions: centres LE stands up and speaks out; champions justice; nurtures connected and collective spaces; mobilises strategically; and leads change.	Peer reviewed journal article
Designated Indigenous Lived Experience roles							
Lee, T., Mckenna, R., Morseau, G., Bacon, A., Baguley, K., Cowdrey‐Fong, S., Bertakis, A., Shorey, T., Smith, T., Aboriginal and Torres Strait Islander Lived Experience Centre Team Mckenna, V., Kitchener, E., Meteoro, N.	2024	Australia	Aboriginal and Torres Strait Islander Lived Experience‐led Peer Workforce Guide (guide)	The guide aims to help Aboriginal and Torres Strait Islander Lived Experience‐led Peer Workers, recognise what their role and responsibilities are in supporting community members, and what they should expect from the organisations they work with.	Co‐Designed/Co‐Created. Used the Aboriginal and Torres Strait Islander Lived Experience Centre's National Network and National Advisory Group to recruit working group members inclusive of diversity who decided on the story and structure of the guide.	Decolonising model setting out the attributes that make a ‘good peer worker’ ‐ scope of practice. Advocacy ‐ connecting to wider systems & ensuring service‐user rights are upheld. Use of Social and Emotional Wellbeing framework (SEWB). Articulates cultural safety, boundaries and cultural considerations.	Framework – grey literature
Walter Wai Tak Chan.	2024	Canada	Psychiatric Consumers and Survivors’ (Lacklustre) Engagement with Indigeneity, Diversity, and Anti‐racist Thought	Identify the extent to which the consumer/survivor/ex‐patient movement (CSX) is engaging with Canadian Indigenous thought.	Qualitative research: interviews with 19 people identifying as being mental health leaders who identified as consumers and survivors and document analysis from 13 organisations.	There is little significant engagement with anti‐racist or Indigenous content in Canada where the research was conducted which could change with increasing the number of Indigenous leaders and engaging in de‐colonising practices.	Peer reviewed journal article
Cooeyate, M. J., Maviglia, M., & Hume, D. (2024).	2024	USA	Developing effective peer support in Native American communities	Identify the value and ingredients of impactful Native American peer support.	A case study on the role and value of peer supervision in the context of designated Native American peer workforce roles.	The paper calls for a strategy around supervision and support for Native American peer workers engaging the combined efforts of policymakers, community organisations, and peer support personnel.	Peer reviewed journal article
Te Hiringa Mahara.	2022	New Zealand	Understanding, honouring and working with ‘Lived Experience’.	This document aims to document and describe the value of Lived Experience workforces, their history and their ongoing growth.	Policy document specifically addressing the history and value of Māori ‘Lived Experience’ workforces	LE communities want LE peers who are in designated LE roles as well as greater and ongoing development of LE leadership. Identifies Māori ‘Lived Experience’ workforces as advocating for equity, an end to coercion and exclusively Western modes of treatment and self‐determination.	Policy – grey literature
Maraku, L.	2024	New Zealand	A Māori Health Workforce with Lived Experience	To profile the Māori lived experience workforce and examine its professional development needs.	A survey conducted with 250 Māori lived experience workers	Results indicated that this workforce felt they had opportunities to advocate and show empathy for others with similar experiences. The research also identified that respondents to the survey regard themselves as having a distinctive professional workforce identity.	Peer reviewed journal article
Easpaig, B.N.G., Lindeman, M.A., Watson, P., Xianliang, L.	2024	Australia	Growing the peer workforce in rural mental health and social and emotional well‐being services: A scoping review of the literature	a)Extent and nature of the literature Providing guidance for growing the peer workforce in rural mental health services; b)identify and explore any guidance relevant to rural peer work services dedicated to First Nations communities, including social and Emotional Wellbeing Frameworks.	A scoping review of the literature where 26 relevant studies were included for review, from the US, UK, Canada and Australia.	When developing Aboriginal and/or Torres Strait Islander lived experience worker positions, it would be important to understand social and emotional well‐being concepts and to be led by local protocols and kinship structures, including what language is used.	Peer reviewed journal article
Epstein, G., Harriott, D., Hermansyne, A., Farah, S., Gold, M., Jennings, L., Nurse, M., Scotton, M., & Walter, J.	2023	Canada	Supporting Peer work	To better understand peer roles, in particular Indigenous peer roles	51 interviews conducted and analysed to answer the questions: how do peers define their roles, and what innovative practices do they engage in as well as suggestions for how to decolonise services.	Peer work was seen to contribute to overall decolonising practices as well as providing necessary and unique services to people.	Grey literature

Table 3 shows the variety of mental health LE roles. Column headings express the type of role with examples within the role type underneath.

**Table 3:** Broad range of mental health lived experience workforce activities.

Management and governance	Advocacy	Education and training	Service design	Service delivery
CEO	Leading advocacy and awareness‐raising	Staff in‐services	Promoting peer support	Establishing peer support
Board Chair	Systemic advocacy	Using media	Promoting recovery	Developing and operating service‐user‐run services
Organisational Board member	Individual advocacy	Working in academia	Programme consultation	Vocational support
Project Officer	Being in a forum	In the community	Programme development	Community inclusion
Programme Manager	Using the media	Orientations	Committee membership	Transitional support with moving from one service programme to another
Discipline lead	Being a committee representative		LE consultancy	Peer support
Team leader				Recovery work
Discipline‐specific supervisor				Drug and alcohol worker
Community of practice facilitator				Group facilitator
Co‐reflection facilitator				

### 3.1 Common Across all Lived Experience Role Categories

The LEW has roots in a socio‐political movement sometimes referred to as the consumer/survivor movement (Beresford & Rose, 2023; Chamberlin, 1990 [3, 5];; Scholz, 2022). LE workers define their discipline and engage in practices that redress people's experiences of being treated as though they are not competent knowers and tellers [21, 22]. Due to direct experience of sense‐making being questioned and disqualified, the LEW tends to practice an openness to multiple world views and ways of making meaning [23]. The ‘power over’ that service users can experience drives the LE workforce to build capability in others [24] and prefer non‐hierarchical decision‐making and power arrangements [3]. Members of the LEW are motivated by emancipation and self‐determination [22]. Furthermore, as LE workers’ identities are often shaped by exclusion [25], practices tend to emphasise inclusiveness and collectivity.

In Australia, the National Lived Experience (Peer) Workforce Development Guidelines [23] state that LE roles are political and rights‐based, connected to systems change and service improvement. Mutuality and connection are valued highly across all role types [11, 23]. They are relational roles [6] where autonomy is highly valued. Evident in the literature was the view that nobody engaging in LE roles should be involved with coercive practices and that this would be anathema to LE ways of working, transgressing an ethical boundary [26]. Some programmes in the US explicitly state that the peer role is a non‐clinical one, and does not involve treatment, assessment or evaluation [17]. The cruciality of discipline specific supervision for all role types was emphasised across the literature as a space in which to gain support and strategise to address unrealistic organisational expectations.

### 3.2 Category One: Lived Experience Direct Service Roles (Such as Peer Support)

By far, the greatest quantity of research literature on the LE workforce focuses on peer support roles. We intentionally selected 10 diverse sources: six peer‐reviewed journal articles, one LE policy Framework from Western Australia, one book chapter, one PhD thesis summary and a brochure detailing peer support competencies.

#### 3.2.1 Peer Support: Role Definition

A person who has gained their insights through life experiences has expertise that is distinct from professional learning [27] and it is this non‐clinical approach that gives peer support its distinctiveness. Peer support is a way of relating where giving and receiving help is mutual, founded on respect, transparency, and shared responsibility. Peer support is not based on psychiatric models and diagnostic criteria [11], but is a real‐world practice of people supporting each other [8] and connecting people to the social, environmental, and political contexts they live in [27].

### 3.3 Knowledge and Direct Service Lived Experience Roles (e.g. Peer Support)

There were three main themes across the literature relating to the knowledge required for direct service LE roles: knowledge that comes from having been there; knowing helpful qualities of relationships; and knowing how to use your own lived experience intentionally.

#### 3.3.1 Knowledge That Comes From Having Been There

The body of knowledge acquired through first‐hand experience of life‐shaping experiences such as discrimination, poverty and oppression, impacts of marginalisation, oppression and loss of autonomy is used to connect, share helpful strategies and validate experiences [17, 28, 29]. Relationships are based on shared lived experience and personal recovery [10, 23]. The ‘embodiment’ of experiential knowledge is the distinctive effect of being supported by someone who has ‘been there’ and shares ideas about ‘how to go on’.

Oborn et al., [28]. Expertise included tacit knowledge about how mental health systems work [28] and being connected to the movement's collective knowledge [30].

#### 3.3.2 Knowing Helpful Qualities of Relationships

Direct service roles are relational [11] By [29, 31, 32]. Making connections, knowing and understanding the value of reciprocity and mutuality are fundamental [10, 12, 29, 31]. These relationships have been described as ‘love labour’ [33]. Focusing on people's strengths was important [10, 33] and integrating experiences rather than being ashamed of them [27]. Understanding trauma and its impact was seen as key knowledge [12, 31]. Knowing the importance of advocacy, choice and control in relationships was highlighted [10, 29, 31, 32].

#### 3.3.3 Knowing How to Use Your Own Lived Experience Intentionally

Knowing the value of explicit use of one's lived experience, being able to think critically, reflect on one's practice and use one's story effectively were seen as important knowledge to have [12, 27, 31, 33]. Being ‘out’ and knowing how to intentionally apply collective peer thinking and values to work was highlighted [29]. One literature review exploring the mechanisms underlying peer support found that because workers have often had unhelpful experiences in their past, they consciously develop approaches that are different from those they received, and this is a key motivator [33]. The same study described these workers being in unique liminal positions occupying two distinct identities as service user and mental health worker.

### 3.4 Practices and Lived Experience Direct Service Roles

There were three main themes across the literature relating to the practices for direct service LE roles: working relationally, sharing LE perspectives in a meaningful way and working in a values and rights‐based way.

#### 3.4.1 Working Relationally

Relationality was seen as foundational in direct service LE work [12, 27] and a willingness to be vulnerable in making a connection [12, 29, 33]. Emotional honesty, authenticity and possessing high levels of emotional understanding were important practices [12, 31, 33]. The ability to use these skills to create and sustain relationships by communicating clearly, connecting through everyday activities, establishing trust, providing practical support, being flexible and responsive to the service user were important practices [12, 17, 27, 28, 31].

#### 3.4.2 Sharing Lived Experience Perspectives in a Meaningful Way

Understanding and supporting the person in distress and sharing one's own insights or perspectives [28] was meaningful and sharing relevant parts of one's own story was a key practice [12, 29, 33]. Being able to transform adverse experiences into powerfully helpful knowledge and skills that can be shared with others was a key skill [12, 29, 33].

#### 3.4.3 Working in a Values and Rights‐Based Way

Individual advocacy for people's preferences, upholding self‐determination and reducing power imbalances were valued practices [12, 31, 32, 34]. On a systems level, being a change agent was valued [12, 31] and focusing on aspects of people's lives rather than clinical diagnoses and symptoms was key [32].

Documentation practices by peer support workers could be uncomfortable. Some peer workers shared minimal information, others did not read notes about people they were working with and some only wrote notes in the service of deepening connection. Prowse [34].

#### 3.4.4 Category Two: Lived Experience Leadership Roles

We sourced seven peer reviewed journal articles focusing primarily on LE leadership, and these in turn, were informed by significant prior scholarship, some of which was theoretical.

There were three main themes across the literature relating to LE leadership roles: knowledge guiding ethical decision‐making; being informed by the ‘collective’ and shared history; having the knowledge to create change.

### 3.5 Knowledge and Lived Experience Leadership Roles

#### 3.5.1 Knowledge Guiding Ethical Decision‐Making

Distinctive leadership knowledge included non‐hierarchical, democratic decision‐making characterised by authentic consultation, shared leadership roles, and creativity [35].

#### 3.5.2 Being Informed By the ‘Collective’ and Shared History

LE leadership roles were connected to and identified with the collective shared history of the movement [1, 29]. Leadership roles used a certain type of power, which was grounded in lived experience, was systems‐informed, and based on knowledge of, and fidelity to, themselves as both individuals and as a collective [22]. Applying, centreing and increasing the influence and availability of LE perspective and knowledge were key [1].

#### 3.5.3 Having the Knowledge to Create Change

Mobilising strategically, and leading change was valued knowledge [35] and achieving systems change or social change were important [1].

### 3.6 Practices and Lived Experience Leadership Roles

LE leadership has been described as an application of expertise rather than an act of representation of others [1]. There were three main themes across our selected literature relating to LE leadership practices: Transforming services from within; embedding the LE workforce and championing justice.

#### 3.6.1 Transforming Services From With in

LE leadership roles engaged in service development, executive level decision‐making [9], policy, governance and management [6, 35]. Better outcomes and improved service quality was created through building relationships, fostering dialogue between service users and service provider perspectives and through being a bridge between them [2, 9]. Leaders could contribute to less restrictive practices through providing more equitable relations between service users and traditional service providers [15, 16, 17]. Embedding LE perspectives into service provision through education and awareness building [15, 17] and activities like implementing surveys on specific topics related to service improvement [9] were raised in the literature.

#### 3.6.2 Embedding the Lived Experience Workforce

Leadership practices included supporting LE workforces by providing line management, discipline and team leadership, planning and developing groups and forums, administration, providing discipline specific supervision and training [9, 17]. Nurturing connected and collective spaces [35] and influencing, collaborating, and promoting a supportive, inclusive culture [1] were highlighted.

#### 3.6.3 Championing Justice

Social justice and human rights concerns underpin LE leadership practices [2, 31]. Standing and speaking up and out were seen as important [35], attending to power imbalances [1] and being a change agent [9]. LE leadership engaged in systemic advocacy [9, 31, 36]. LE leadership also involved research and information gathering, and having to legitimise their roles [17].

### 3.7 Category Three: Designated Indigenous Lived Experience Roles

There are comparatively few designated Indigenous LE roles in mental health services across the four countries represented in the literature. Our review included seven resources: two each from Canada (Turtle Island), Australia and New Zealand (Aotearoa) and one from a Native American perspective (Turtle Island). There are contextual differences between countries, such as Australia's Aboriginal and Torres Strait Islander experiences with Terra Nullius (‘land of no people’) and Aotearoa, for instance, with its Treaty of Waitangi. However, descriptions of experiences, knowledge and practices displayed by a mental health Indigenous LE worker across the four countries were very similar. Notably, Indigenous populations have been subjected at disproportionate levels to mental health laws and have experienced greater levels of coercion in mental health services than non‐Indigenous populations (Te Hiringa [37]). There were three main themes across the literature concerning important knowledge needed for designated Indigenous roles in mental health services: challenging exclusively Euro‐centric modes of treatment, truth‐telling and self‐determination.

### 3.8 Knowledge and Designated Indigenous Lived Experience Roles

#### 3.8.1 Challenging Exclusively Euro‐Centric Modes of Treatment

Understanding that the exclusivity of Euro‐centric models of mental health could undermine Indigenous wellbeing and challenging this were seen as important knowledge [38]; (Te Hiringa [37]). Traditional ways of healing and a decolonising focus on healing, cultural values, honouring Indigenous ways of knowing, being and doing was featured [4, 38].

#### 3.8.2 Truth‐Telling

Recognising colonisation, institutionalised racism, unconscious bias, interpersonal violence and micro‐aggressions and their impact was important knowledge [38]; (Te Hiringa [37]). Knowing the impacts of intersections of social determinants of health, including poverty, unemployment, housing insecurity and inadequate access to health care as well as how discrimination and racism compound these challenges was highlighted [4, 38]. Understanding the historical and ongoing effects of colonisation and building in de‐colonising approaches was valued knowledge and from this vantage point, knowledge of the impact of social, political and historical determinants was essential [4, 38]; (Te Hiringa [37]). Intersecting lines of power, discrimination and oppression impacted designated Indigenous LE workers, and the roles they played could be affected by inappropriate, Western ideas about ‘professionalism’ [39].

#### 3.8.3 Self‐Determination

Necessary knowledge for designated Indigenous LE roles centred on self‐determination [4]. Ending coercion, valuing alternatives, knowing about equity and human rights informed approaches were key Te Hiringa [37]. Knowing how to centre self‐determination and the community was important knowledge [4].

### 3.9 Practices and Designated Indigenous Lived Experience Roles

There were three main themes emerging from the literature about practices of designated Indigenous LE roles: relational and community approach, trauma‐informed, culturally sensitive and safe.

#### 3.9.1 Relational and Community Approach

A dissimilar role scope to other LE roles is that relational approaches of designated Indigenous roles in mental health often involve interpersonal, family and community levels as part of their scope, whereas non‐Indigenous LE roles might focus more on individuals or groups [38]. Story‐telling and yarning are ways of relating and there is an expectation that relationships should invoke reciprocity and connection [4]. Indigenous LE workers take people as they are (Lee et al., 2004 [38]:). Collective decision‐making is a featured practice [4].

#### 3.9.2 Trauma Informed

Understanding that individuals may incur single incidents of, or cumulative marginalisation and how to respond to this is valued particularly in contexts of colonisation and settler violence [4, 38].

### 3.10 Culturally Sensitive and Safe

Indigenous LE workers sought to remove power imbalances, understand impacts of Intersectionality on identities, and ensure the service user defines what is culturally safe for them [4]. In terms of their own practice, Indigenous LE workers needed to be able to exercise autonomy [4]. Culturally sensitive ways of working were necessary for the work to be impactful [38].

**Figure 2:**
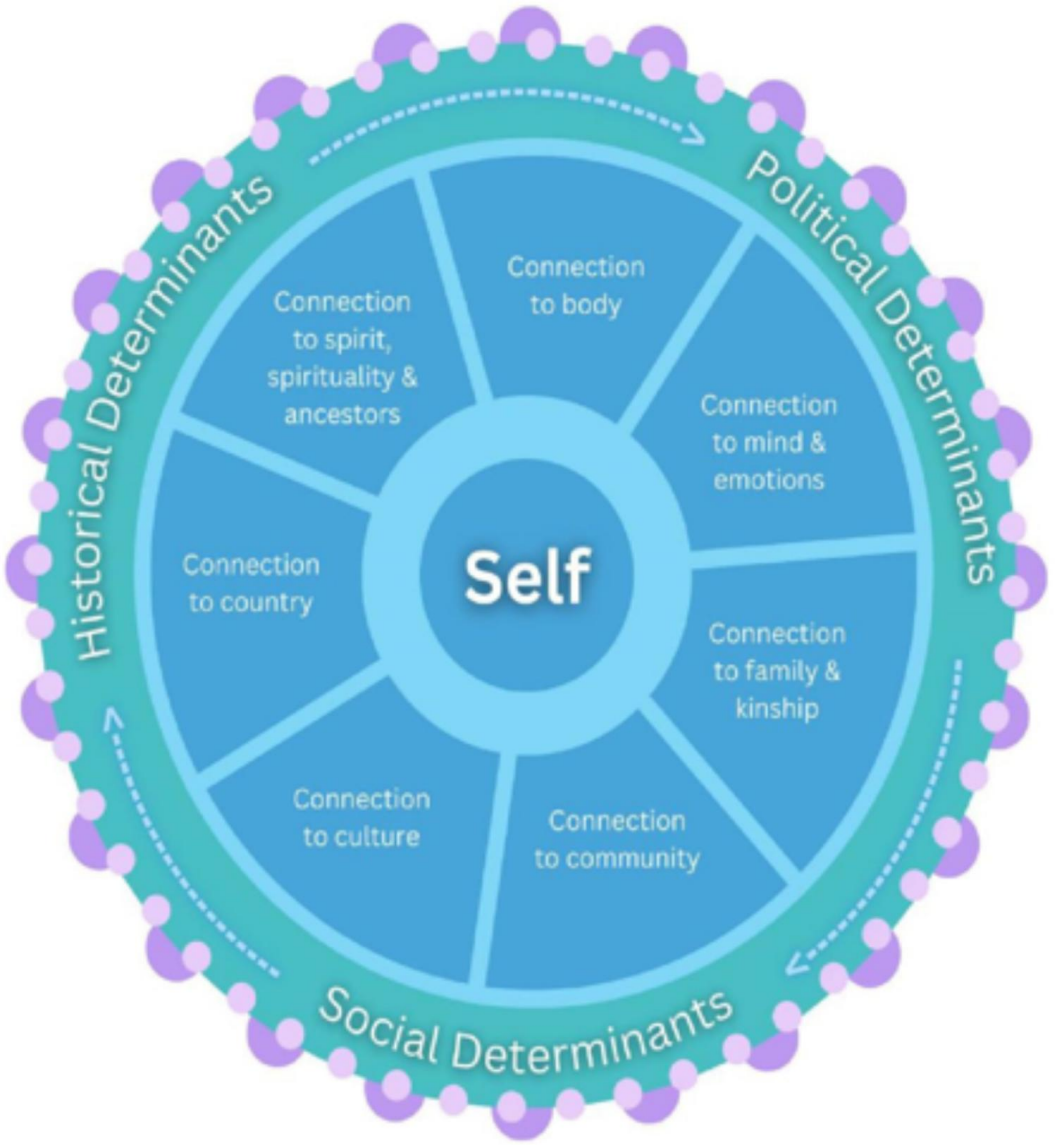
The social and emotional wellbeing approach adapted from Gee et al. [40].

Figure 2 depicts a non‐Eurocentric wellbeing lens that was developed to demonstrate the interaction of connection domains and social determinants for Aboriginal and Torres Strait Islander people. In Western Australia, this Framework informs the designated Indigenous LE workforce [4]. There is evident potential for Indigenous leadership informing the further development of the LE workforce, mental health professionals and mental health services with its focus on wellbeing, relationality, upholding self‐determination, de‐pathologising, recognising human rights and as a response to colonial violence.

## 4 Discussion

Comparatively, there is more literature describing principles guiding LE work than descriptions of practices. The key contribution from the 25 resources we analysed across five English‐speaking countries in this narrative review revealed that the LE workforce has been thinking about and articulating its knowledge and practices as a discipline. Many papers simultaneously explored the difficulties of preserving the distinctive nature of this non‐clinical, human rights‐informed discipline within clinical contexts.

Over the past two decades, a growing body of evidence highlights the benefits of the LEW [27, 41]. In many countries, its value is now accepted in policy and is recognised as an important part of the configuration of service provision [6]. For example, research into peer support finds it provides recovery outcomes that are at least equivalent to usual care conditions and/or services provided by non‐peer staff [41]; enables recovery [8]; and effectively challenges stigma [18]. Research shows the LEW improves the culture, quality, effectiveness, and responsiveness of services [9]. Organisations benefit from the impact, perspective and leadership of the LEW in social policy, system management, planning, education, programme development, and evaluation [2, 13]. Despite these benefits, the roles are often poorly understood, leaving them vulnerable to co‐option.

### 4.1 Avoiding Co‐Option

Research has recommended LE workers need to set their own parameters of what constitutes the LE discipline is and how this workforce should be described, rather than these phenomena being described by others [18]. Policy and governance contexts for paid LE work are critical, and commitments to LEW involvement in decision‐making within a service are a key success factor [42]. Roennfeldt, Byrne [6]. Other effective actions include: clear position descriptions and adequate training; career pathways; supportive measures such as reasonable adjustments and flexibility; organisational culture and commitment; senior roles for LE worker; allies and champions of LE roles within organisations and organisations who have had success embedding LE workforce sharing their expertise with other organisations [16].

Other strategies for avoiding co‐option, are opportunities for this workforce to be grounded in its history, values and civil rights/social justice frameworks have been suggested [6, 36]. Co‐reflection, discussing experiences, and debriefing with peer colleagues are other strategies [34]. Refusing to engage in work that does not align with LE values and pointing to relevant articles or Frameworks for justification have also been put forward [34]. Connecting politically with the consumer/survivor/Mad movement and reading LE authored literature has been suggested [30]. Resourcing of LE specific spaces, access to LE provided training, providing access to all staff about LE perspectives, and thus influencing decision making are other essential infrastructures for LE role development [43].

Understanding the significance of intersectionality and diversity in terms of identity and social and cultural contexts [26] and awareness‐raising around how power operates [30] have been put forward as important. The need to be aware of tensions of roles that may reproduce systems of oppression has been identified [30]. The value of resistance, consciousness of both constraints and opportunity and the recognition of these as systemic rather than personal failings are also identified as positive steps that the LE workforce can take [30].

To this we would add the importance of learning from Indigenous leadership in policy and systems advocacy as valuable foundations for all LE work [4]. Consumer movements and organisations have been found lacking in terms of incorporating Indigenous thought and leadership (Chan, 2024). Low workforce numbers have impacted the extent to which Indigenous LE leadership has been able to influence the broader LE workforce, other mental health professional roles and mental health services. Despite being considered invaluable to the range of mental health workforces, more research is needed to explore what organisations can do to understand and implement Indigenous leadership (Maraku Te Rau Ora, 2024; Nic Giolla [44]; Te Hiringa [37]).

### 4.2 Limitations

We acknowledge the limitations of a narrative review in that it is not intended to be a replicable systematic search and analysis. On the other hand, the research team had significant content expertise, aiding the sourcing of material. To some extent we use the Australian LE workforce as a starting point and this may not exactly reflect the experience of other jurisdictions, however our literature review reveals similar experiences were encountered in other countries. Our searches relevant to Indigenous LE workforces will likely not have captured the full range and diversity of roles, underpinning knowledge and practices. As Australian LE researchers, it is likely that we missed terminology that would be commonly used in other countries.

Our narrow focus on the LE workforce within mental health service systems would entail missing insights gathered from such settings as independently run community groups or LE‐led alternatives. Our findings are also unlikely to illustrate how, for example, intersectionality and structural inequalities impact specialised LE workforce roles such as people who are culturally and racially marginalised, young people, aged, forensic, dual disability, or LGBTIQ+ (Mental Health Commission, 2021). We did not undertake critical appraisal in this study, meaning the quality of the evidence was not determined. Further systematic research could be undertaken to achieve this.

## 5 Conclusion

An understanding within mental health services of the distinctive knowledge and practices underpinning LE roles is vital to their ongoing effectiveness. When well supported, and with opportunities for leadership and senior positions, the LE workforce can engage in much needed systems advocacy, and provision of distinctive non‐clinical, rights‐based services. Insights from the LEW itself are essential in an environment where rapid workforce growth could otherwise miss the mark.

By examining seminal and contemporary literature framing the LE discipline, this narrative review highlighted how members of the LEW describe and enact our discipline. The weight of evidence we reviewed defines the knowledge and practices for three main types of LE roles from the vantage point of those engaging in the workforce: direct service provision such as peer support, leadership and designated Indigenous LE roles. We position Indigenous LE leadership as vital to strengthening the rights‐informed, non‐clinical LEW roles because of their focus on wellbeing, non‐pathologisation, self‐determination, and social determinants and because this is a necessary response to ongoing colonial violence.

## Patient or Public Contribution

This research was lived experience‐led. Seven of the researchers undertaking this narrative study were service user academics, meaning, they had experience of using mental health services and were also working within a University setting, on a variety of research and other projects. One nurse academic provided research supervision. The lived experience researchers led the design, conduct, analysis and interpretation of the data and prepared the manuscript.

## Author Contributions

**Cath Roper:** conceptualisation, writing – original draft, investigation, methodology, writing – review and editing, data curation, formal analysis. **Shibs Sharpe:** conceptualisation, investigation, writing – original draft, writing – review and editing. **Leanna Azoury:** Conceptualisation, Investigation, Writing – original draft, Methodology, Writing – review and editing, Data curation. **Nina Joffee‐Kohn:** conceptualisation, investigation, writing – original draft, writing – review and editing, project administration, software, formal analysis, visualisation. **Natasha Swingler:** conceptualisation, writing – original draft, writing – review and editing. **Puneet Sansanwal:** writing – original draft, investigation. **leah mckenner:** writing – original draft, investigation. **Cat Van Remmen:** conceptualisation, investigation. **Bridget Hamilton:** supervision, writing – review and editing, methodology, writing – original draft, conceptualisation, formal analysis, validation.

## Ethics Statement

Ethics was not required for the conduct of this study. The Social and Emotional Wellbeing wheel has permission to be freely used. Patient consent statements were not required for this research.

## Conflicts of Interest

The authors declare no conflicts of interest.

## Data Availability

No new data was generated by this narrative review and all data analysed was from publicly available sources, or data from studies that can be requested from the original authors. All the resources included in this review can be readily located.
